# COVID-19 vaccine uptake among people with HIV: identifying characteristics associated with vaccine hesitancy

**DOI:** 10.1038/s41598-023-47106-8

**Published:** 2023-11-23

**Authors:** Karol Boschung, M. John Gill, Hartmut B. Krentz, Jessica Dalere, Brenda Beckthold, Kevin Fonseca, Jeffrey A. Bakal, Jacqueline M. McMillan, Jamil Kanji, Raynell Lang

**Affiliations:** 1 Department of Medicine, University of Calgary, Calgary, AB Canada; 2 Southern Alberta Clinic, Calgary, AB Canada; 3 Department of Community Health Sciences, University of Calgary, Calgary, AB Canada; 4 Alberta Public Health Laboratory, Alberta Precision Laboratories, Calgary, AB Canada; 5 Department of Microbiology, Immunology and Infectious Diseases, University of Calgary, Calgary, AB Canada; 6 Provincial Research Data Services, Alberta Health Services, Calgary, AB Canada; 7 Section of Medical Microbiology, Department of Pathology and Laboratory Medicine, University of Calgary, Calgary, AB Canada; 8 Department of Laboratory Medicine and Pathology, Faculty of Medicine and Dentistry, University of Alberta, Edmonton, AB Canada

**Keywords:** HIV infections, Infectious diseases, Viral infection, Epidemiology

## Abstract

People with HIV (PWH) are at increased risk of COVID-19 infection. Both Canadian (NACI) and US (CDC) guidelines recommend that all PWH receive at least 2 doses of COVID-19 vaccine, and a booster. We examined vaccination uptake among PWH in Southern Alberta, Canada. Among adult PWH, we evaluated COVID-19 vaccination uptake between December 2020 and August 2022. Poisson regression models with robust variance (approximating log binomial models) estimated crude and adjusted prevalence ratios (aPR) and 95% confidence intervals (CI) for receiving (1) any vs. no vaccine, and (2) primary series with booster (≥ 3 vaccines) versus primary series without booster. Among 1885 PWH, 10% received no COVID-19 vaccinations, 37% < 3 vaccines and 54% received ≥ 3 vaccines. Females (vs. males) were less likely to receive a vaccine booster. Receiving no COVID-19 vaccines was associated with White ethnicity, unsuppressed HIV viral load (> 200 copies/mL), and using illegal substances. Factors associated with decreased booster uptake included being younger, Black (vs. White) ethnicity, substance use, lower educational attainment, and having an unsuppressed HIV viral load. COVID-19 booster uptake among PWH does not meet vaccine guidelines, and receipt of vaccines is unevenly distributed. Booster uptake is lowest among young females and marginalized individuals. Focused outreach is necessary to close this gap.

## Introduction

Vaccination is crucial to the care of people with HIV (PWH), who are at a greater risk for several vaccine preventable illnesses^1,2^. Both the Canadian National Advisory Committee on Immunization (NACI) and the United States Centers for Disease Control and Prevention (CDC) guidelines recommend that all PWH receive a primary series of SARS-CoV-2 vaccines with either 2 or 3 mRNA vaccine doses (depending on the individual’s CD4 count and HIV treatment status) followed by a booster dose^3–5^. Studies have documented an ongoing increased risk of breakthrough SARS-CoV-2 infection despite vaccination among PWH compared to people without HIV^6^. However, despite this elevated risk, vaccine hesitancy (VH) towards COVID-19 vaccines is common^7–9^. Therefore, understanding the drivers of COVID-19 vaccination amongst PWH is needed to develop targeted strategies to increase uptake.

While VH has long been a subject of concern to health practitioners^10^, the SARS-CoV-2 pandemic has highlighted the pressing need to understand this growing movement. VH is multifactorial, with several models being proposed to explain differential vaccine uptake, such as the “3C” (Complacency, Confidence, Convenience) model^11^. These models demonstrate that vaccination rates are driven not only by individual beliefs, but also by larger social and demographic factors. For example, while negative beliefs about the efficacy or safety of vaccination have been linked to low uptake^9,12–16^, higher rates of vaccination have also been consistently correlated with increased age, both in PWH^9,12,17–22^ and non-PWH^14,23,24^ populations. Similarly, in PWH, lower vaccination rates have been associated with lower socioeconomic status (SES)^25^, unemployment and incarceration^26^, and lower educational attainment^27^. Laboratory measures of HIV care engagement, such as CD4 count and HIV viral load (VL), have also been found to predict vaccination rates among PWH, with lower CD4 count and unsuppressed HIV VL generally being correlated with lower vaccination rates, including against SARS-CoV-2^8,17,19,22,28–31^. This highlights the complex associations of VH and vaccine uptake.

We aimed to assess initial vaccine uptake (both any vaccine and booster doses following a primary series of vaccine) against SARS-CoV-2 among PWH in Southern Alberta, Canada and then determine associated demographic, social, and clinical factors.

## Methods

### Study design and study population

**Figure 1:**
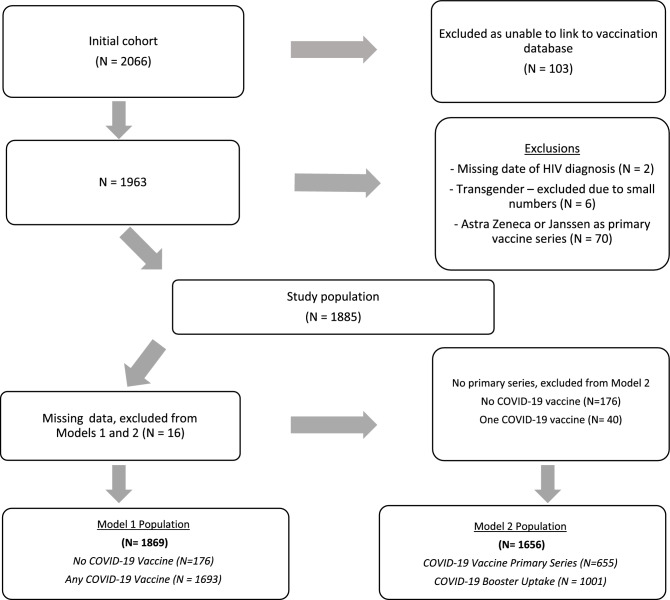
Inclusion and exclusion criteria for the study population.

All PWH in southern Alberta, Canada receive their HIV care through a centralized program at the Southern Alberta Clinic (SAC). HIV care is publicly funded and free of charge. SAC has a comprehensive, longitudinal database that collects demographic, clinical and laboratory data on all cohort participants^32^. We conducted a retrospective cohort study of all adult PWH (≥ 18 years) in care at SAC as of June 1, 2022 (N = 2066). Inclusion/exclusion criteria are detailed in Fig. 1. Due to concerns of heterogeneity and different booster guidelines, those who received the AstraZeneca or Janssen COVID-19 vaccine as their primary vaccine dose were excluded (N = 70). The use of non-nominal SAC cohort data for research has been approved by the University of Calgary Conjoint Health Research Ethics Board along with the current research protocol (REB22-1071), under waiver of consent. All methods were performed in accordance with relevant guidelines and regulations.

### COVID-19 vaccination ascertainment

Vaccine uptake was measured between December 2020 and August 2022, to match vaccine availability and boosting protocols in Alberta, Canada. COVID-19 vaccination data was obtained through linkage to the provincial immunization database. Booster dosing was defined as having received 3 or more vaccines against SARS-CoV-2. This is a conservative estimate of booster dosing for some PWH, as NACI and CDC guidelines recommend that PWH with severe immune suppression (such as CD4 count ≤ 200 cells/mm^3^) receive a three dose primary series, with the booster as fourth dose^4,5^. However, due to guideline modifications during the study period with regards to the timing of booster administration vs extended (three-dose) primary series, for the purposes of this study all patients with ≥ 3 doses were defined as having received a booster dose. Care providers at SAC encourage vaccine uptake, however COVID-19 vaccines are not provided at the clinic. COVID-19 vaccines were freely available during the study period at many pharmacies, primary care offices, and health centres across the province.

### Covariates

Sex was defined as sex assigned at birth. Race/ethnicity was self-reported and categorized as White, First Nations (Indigenous/Métis), Black, and Other (Hispanic, East-Asian, West-Asian, Indo-Asian, other). HIV acquisition risks were categorized as people who inject drugs (PWID), gay/bisexual/men who have sex with men (gbMSM), heterosexual, or other (blood transfusion or perinatal transmission). Age as of June 1, 2022 was included. Comorbidities were determined from the electronic medical record (EMR) as of June 1, 2022, and included the following diagnoses: hypertension, diabetes mellitus, renal disease, malignancy (excluding non-melanoma skin cancer), asthma/COPD or cardiovascular disease or BMI ≥ 25. Most recent body mass index (BMI) was categorized if they had a measurement between January 1, 2019 and June 1, 2022. Other covariates obtained from the medical record at study baseline included highest recorded education level, history of incarceration, history of homelessness or unstable housing, history of substance use (excluding Marijuana use), and CD4 nadir. Most recent place of residence was defined as metropolitan (city of > 1 million people) vs non-metropolitan (< 1 million people). Time-varying covariates included CD4 count and HIV VL, measured during the study period. If multiple measures were available, we used the lowest CD4 count and highest VL recorded during the study period.

### Analysis

Descriptive analyses were performed using chi-squared tests for categorical variables and t-tests for continuous variables to identify factors associated with COVID-19 vaccine uptake. Uptake was categorized as no vaccine, 1–2 doses of vaccine and ≥ 3 doses of vaccine. Observations with missing data (N = 16, 1%) were excluded from regression analysis. Poisson regression models with robust variance (approximating log binomial models) estimated crude and adjusted prevalence ratios (aPR) and 95% confidence intervals (CI) for (1) PWH who received any COVID-19 vaccine compared with no vaccine and (2) PWH who received ≥ 3 COVID-19 vaccines (booster dose) compared to those who received 2 doses of COVID-19 vaccines (a primary series). Models were adjusted for age as of June 1, 2022, sex, race/ethnicity, HIV acquisition risk, highest education level, lowest CD4 count and highest viral load measure over vaccination period based on a priori literature review. Other covariates included in the models were based on backwards stepwise regression including metropolitan location and substance use history for Model 1, and history of incarceration, BMI category, substance use and comorbidities for Model 2. All p-values are two-tailed tests with the statistical significance level set at *p* = 0.05 including 95% confidence intervals. Analyses were performed using STATA version 17.0 (College Station, TX).

## Results

### Patient characteristics

**Figure 2:**
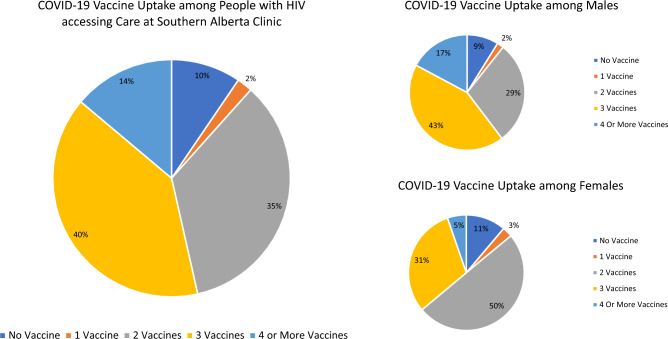
COVID-19 vaccine uptake by sex among people with HIV active in care at Southern Alberta Clinic.

Among 1885 PWH receiving care at SAC as of June 1, 2022, 179 (10%) had received no COVID-19 vaccination, 40 (2%) had received one dose, 657 (35%) two doses, 748 (40%) three doses and 261 (14%) four or more doses (Fig. 2). Therefore, 1,666 (88%) had received at least a primary series and 1009 (54%) had received at least three doses of COVID-19 vaccine and were considered as having received a booster dose. Half of the study population (50%) were ≥ 50 years old, with a median age of 50 years (IQR 42-58) (Table 1). The majority were male (72%), 47% self-identified as White and 29% as Black. The main HIV acquisition risk factors identified in the study population were heterosexual risk (44%) and MSM/Bisexual (42%) with 8% reporting injection drug use. Over the study period, few participants (6%) had CD4 counts < 200 cells/mm^3^ and 7% had VL $\ge$ 200 copies/mL.

**Table 1:** Description of the characteristics of the study population by COVID-19 vaccine uptake.

Characteristics	Total	No vaccine	2 or Less vaccines	3 or More vaccines^A^
	N = 1885	N = 179 (10%)	N = 697 (37%)	N = 1009 (54%)
Age (median, IQR)	50.1 (41.5–58.3)	47.2 (40.5–57.7)	46.0 (38.6–52.5)	54.0 (43.9–61.4)
< 40 years	406 (22%)	42 (23%)	204 (29%)	160 (16%)
40–50 years	531 (28%)	56 (31%)	254 (36%)	221 (22%)
50–60 years	564 (30%)	51 (28%)	180 (26%)	333 (33%)
> 60 years	384 (20%)	30 (17%)	59 (8%)	295 (29%)
Birth sex				
Male	1356 (72%)	120 (67%)	418 (60%)	818 (81%)
Female	529 (28%)	59 (33%)	279 (40%)	191 (19%)
Ethnicity/race				
Non-Hispanic white	893 (47%)	95 (53%)	206 (30%)	592 (59%)
Non-Hispanic black	542 (29%)	48 (27%)	325 (47%)	169 (17%)
First nations	117 (6%)	12 (7%)	57 (8%)	48 (5%)
Other/unknown^B^	333 (18%)	24 (13%)	109 (16%)	200 (20%)
HIV acquisition risk category				
MSM/Bisexual	782 (41%)	47 (26%)	166 (24%)	569 (56%)
Heterosexual	835 (44%)	94 (53%)	419 (60%)	322 (32%)
PWID	148 (8%)	23 (13%)	56 (8%)	69 (7%)
Other/unknown^C^	120 (6%)	15 (8%)	56 (8%)	49 (5%)
Nadir CD4 category				
> 500 cells/mm^3^	175 (9%)	23 (13%)	71 (10%)	81 (8%)
200–500 cells/mm^3^	857 (45%)	79 (44%)	328 (47%)	450 (45%)
< 200 cells/mm^3^	849 (45%)	75 (42%)	297 (43%)	477 (47%)
Missing	4 (0%)	2 (1%)	1 (0%)	1 (0%)
CD4 category				
> 500 cells/mm^3^	880 (47%)	65 (36%)	309 (44%)	506 (50%)
200–500 cells/mm^3^	682 (36%)	53 (30%)	252 (36%)	377 (37%)
< 200 cells/mm^3^	115 (6%)	12 (7%)	59 (8%)	44 (4%)
Not measured over study period	208 (11%)	49 (27%)	77 (11%)	82 (8%)
Viral load category				
< 200 copies/mL	1635 (87%)	111 (62%)	580 (83%)	944 (94%)
≥ 200 copies/mL	137 (7%)	30 (17%)	72 (10%)	35 (3%)
Not measured over study period	113 (6%)	38 (21%)	45 (6%)	30 (3%)
Ever incarcerated	110 (6%)	15 (8%)	52 (7%)	43 (4%)
Initial vaccine type				
Moderna	409 (22%)	0 (0%)	195 (28%)	214 (21%)
Pfizer-BioNTech	1297 (69%)	0 (0%)	502 (72%)	795 (79%)
Education level				
< 12 years	352 (19%)	44 (25%)	172 (25%)	136 (13%)
12 years	426 (23%)	47 (26%)	169 (24%)	210 (21%)
> 12 years	906 (48%)	55 (31%)	264 (38%)	587 (58%)
Missing/unknown	201 (11%)	33 (18%)	92 (13%)	76 (8%)
Location^D^				
Metropolitan	1495 (79%)	126 (70%)	566 (81%)	803 (80%)
Non-Metropolitan	374 (20%)	50 (28%)	126 (18%)	198 (20%)
Missing	16 (1%)	3 (2%)	5 (1%)	8 (1%)
Housing status				
Unstable housing/homeless	182 (10%)	24 (13%)	74 (11%)	84 (8%)
Body mass index				
< 25 kg/m^2^	544 (29%)	57 (32%)	190 (27%)	297 (29%)
≥ 25 kg/m^2^	1034 (55%)	85 (47%)	354 (51%)	595 (59%)
Missing	307 (16%)	37 (21%)	153 (22%)	117 (12%)
History of substance use	261 (14%)	36 (20%)	93 (13%)	132 (13%)
Comorbidities				
Malignancy	155 (8%)	12 (7%)	35 (5%)	108 (11%)
Hypertension	240 (13%)	17 (9%)	64 (9%)	159 (16%)
Renal disease	61 (3%)	4 (2%)	15 (2%)	42 (4%)
Cardiovascular disease	64 (3%)	4 (2%)	12 (2%)	48 (5%)
Asthma or COPD	201 (11%)	19 (11%)	55 (8%)	127 (13%)
Diabetes mellitus	163 (9%)	7 (4%)	54 (8%)	102 (10%)

IQR—Interquartile range, MSM—men who have sex with men, PWID—people who use injection drugs, COPD—chronic obstructive pulmonary disease.

^A^Booster dose defined as receiving 3 or more COVID-19 vaccinations.

^B^Other Ethnicity/Race includes Hispanic, East-Asian, West-Asian, Indo-Asian, or other.

^C^Other HIV risk acquisition group includes blood transfusion, hemophilia or perinatal transmission.

^D^Location defined as most recent place of residence, metropolitan and non-metropolitan (city of > 1 million people) or rural (< 1 million people).

### Characteristics of PWH who had not received any COVID-19 vaccine

Among the 179 PWH without any COVID-19 vaccination a greater proportion were female, White, or with injection drug use or heterosexual sex reported as their primary HIV risk factor (Table 1). A greater proportion of PWH who had not received a COVID-19 vaccine also had no CD4 count measurements (27% vs. 9%) or HIV VL (21% vs. 4%) measurements during the study period. Those with no COVID-19 vaccination were also more likely to have ≤ 12 years of education, have ever been incarcerated, reported unstable housing, a history of substance use, or live in a non-metropolitan location.

### Factors associated with not receiving COVID-19 vaccine (vs. receiving at least one dose)

In adjusted analyses, age and sex were not significantly associated with receiving no COVID-19 vaccine (Table 2). Compared with White PWH, Black PWH were 8% more likely to have received any COVID-19 vaccine (aPR 1.08 [95% CI 1.03–1.13]). PWH who reported heterosexual sex or PWID were less likely to receive any COVID-19 vaccine compared with PWH reporting gbMSM HIV acquisition risks.

There were no significant associations with vaccine uptake by CD4 count, however PWH with VL measures $\ge$ 200 copies/mL were 15% less likely to be vaccinated (vs. PWH with VL measures < 200 copies/mL) (aPR 0.85 [95% CI 0.78–0.94]). PWH without a VL measurement over the study period were less likely to receive COVID-19 vaccination (aPR 0.76 [95% CI 0.66–0.87]). In unadjusted analyses, living in a non-metropolitan (vs. metropolitan) location was associated with a 5% reduced likelihood for any COVID-19 vaccination, however this attenuated to a statistically non-significant 3% reduction following adjustment. A history of ever using substances (vs. never using substances) was associated with a 4% reduced likelihood of receiving any COVID-19 vaccination (aPR 0.96 [95% CI 0.91–1.00]). Besides Diabetes Mellitus, which was associated with a 6% increased likelihood of receiving at least one COVID-19 vaccine in unadjusted analyses (PR 1.06 [95% CI 1.02–1.10]), all other assessed comorbidities were not associated with any (vs. no) vaccine uptake.

**Table 2:** Crude and adjusted prevalence ratios for COVID-19 booster uptake among People with HIV at Southern Alberta Clinic.

Characteristics	Model 1: Likelihood of accepting any COVID-19 vaccine (N = 1693) versus no COVID-19 vaccines (N = 176)				Model 2: Likelihood of accepting a COVID-19 booster vaccine (N = 1001) versus primary series (N = 655)^A^			
	Crude prevalence ratio (95% CI)	*P*-value	Adjusted prevalence ratio* (95% CI)	*P*-value	Crude prevalence ratio (95% CI)	*P*-value	Adjusted prevalence ratio** (95% CI)	*P*-Value
Age								
< 40 years	Ref		Ref		Ref		Ref	
40–50 years	1.00 (0.96–1.04)	0.961	1.00 (0.96–1.04)	0.979	1.07 (0.92–1.24)	0.365	**1.16 (1.01–1.32)**	**0.032**
50–60 years	1.01 (0.97–1.05)	0.615	1.01 (0.96–1.05)	0.810	**1.47 (1.30–1.68)**	**< 0.001**	**1.39 (1.22–1.57)**	**< 0.001**
> 60 years	1.03 (0.98–1.07)	0.261	1.02 (0.97–1.06)	0.477	**1.89 (1.67–2.13)**	**< 0.001**	**1.61 (1.42–1.82)**	**< 0.001**
Birth sex								
Male	Ref		Ref		Ref		Ref	
Female	0.97 (0.94–1.01)	0.165	1.01 (0.97–1.06)	0.507	**0.62 (0.55–0.70)**	**< 0.001**	0.91 (0.80–1.03)	0.145
Ethnicity/race								
Non–Hispanic White	Ref		Ref		Ref		Ref	
Non–Hispanic Black	1.02 (0.98–1.05)	0.302	**1.08 (1.03–1.13)**	**0.003**	**0.46 (0.41–0.53)**	**< 0.001**	**0.63 (0.54–0.73)**	**< 0.001**
First nations	1.01 (0.95–1.08)	0.744	**1.10 (1.02–1.31)**	**0.015**	**0.64 (0.51–0.78)**	**< 0.001**	0.88 (0.78–1.07)	0.193
Other/unknown^B^	1.04 (1.00–1.08)	0.066	**1.05 (1.01–1.09)**	**0.030**	**0.87 (0.79–0.95)**	**0.002**	1.03 (0.94–1.13)	0.549
HIV acquisition risk category								
MSM/Bisexual	Ref		Ref		Ref		Ref	
Heterosexual	**0.94 (0.91–0.97)**	**< 0.001**	**0.91 (0.87–0.96)**	**< 0.001**	**0.57 (0.52–0.62)**	**< 0.001**	**0.81 (0.72–0.91)**	**< 0.001**
PWID	**0.90 (0.83–0.96)**	**< 0.001**	0.93 (0.86–1.00)	0.056	**0.74 (0.63–0.86)**	**< 0.001**	0.91 (0.78–1.07)	0.276
Other/unknown^C^	**0.93 (0.86–0.99)**	**0.009**	**0.92 (0.85–0.99)**	**0.024**	**0.62 (0.50–0.76)**	**< 0.001**	0.92 (0.75–1.13)	0.424
CD4 category								
> 500 cells/mm^3^	Ref		Ref		Ref		Ref	
200–500 cells/mm^3^	0.99 (0.97–1.02)	0.726	1.00 (0.97–1.03)	0.865	0.98 (0.90–1.06)	0.574	0.99 (0.92–1.07)	0.847
< 200 cells/mm^3^	0.97 (0.91–1.04)	0.404	1.04 (0.97–1.11)	0.282	**0.69 (0.54–0.87)**	**0.002**	**0.79 (0.65–0.96)**	**0.019**
No measurement	**0.83 (0.76–0.89)**	** < 0.001**	0.94 (0.88–1.01)	0.077	**0.87 (0.74–1.02)**	**0.080**	0.95 (0.81–1.10)	0.489
Viral load category								
< 200 copies/mL	Ref		Ref		Ref		Ref	
≥ 200 copies/mL	**0.85 (0.77–40.92)**	**< 0.001**	**0.85 (0.78–0.94)**	**0.001**	**0.53 (0.41–0.70)**	**< 0.001**	**0.69 (0.53–0.91)**	**0.009**
No measurement	**0.71 (0.62–0.81)**	**< 0.001**	**0.76 (0.66–0.87)**	**< 0.001**	**0.71 (0.54–0.93)**	**0.014**	0.89 (0.67–1.19)	0.435
Incarcerated								
Never	Ref		–		Ref		Ref	
Ever	0.95 (0.88–1.02)	0.182	–		**0.78 (0.62–0.97)**	**0.028**	0.87 (0.69–1.10)	0.240
Education level								
< 12 years	Ref		Ref		Ref		Ref	
12 years	1.02 (0.96–1.07)	0.544	1.00 (0.95–1.06)	0.938	**1.23 (1.06–1.43)**	**0.003**	**1.16 (1.01–1.33)**	**0.034**
> 12 years	**1.07 (1.03– 1.12)**	**0.001**	1.04 (0.99–1.09)	0.132	**1.51 (1.33–1.73)**	**< 0.001**	**1.20 (1.06–1.36)**	**0.005**
Unknown	0.96 (0.89–1.03)	0.284	0.95 (0.88–1.03)	0.212	1.00 (0.81–1.23)	0.992	1.06 (0.88–1.29)	0.522
Location^D^								
Metropolitan	Ref		Ref		Ref		–	
Non-Metropolitan	**0.95 (0.91–0.99)**	**0.011**	0.97 (0.93–1.01)	0.183	1.05 (0.95–1.15)	0.327	–	
Housing status								
Stable housing	Ref		–		Ref		–	
Unstable housing/homeless	0.95 (0.88–1.02)	0.182	–		0.92 (0.80–1.07)	0.295	–	
Body mass index								
< 25 kg/m^2^	Ref		–		Ref		Ref	
≥ 25 kg/m^2^	1.03 (0.99–1.06)	0.150	–		1.02 (0.94–1.11)	0.680	1.05 (0.98–1.14)	0.169
Measurement missing	0.99 (0.94–1.04)	0.629			**0.70 (0.60–0.82)**	**< 0.001**	0.87 (0.75–1.01)	0.072
History of substance use								
Never	Ref		Ref		Ref		Ref	
Ever	**0.94 (0.90–0.99)**	**0.026**	**0.96 (0.91–1.00)**	**0.049**	1.03 (0.92–1.15)	0.629	**0.90 (0.81–0.99)**	**0.033**
Comorbidities (vs. without these conditions)								
Malignancy	1.02 (0.97–1.07)	0.416	–	0.889	**1.31 (1.18–1.44)**	**< 0.001**	1.02 (0.93–1.12)	0.620
Hypertension	1.03 (0.99–1.07)	0.149	–	0.907	**1.26 (1.15–1.38)**	**< 0.001**	1.03 (0.94–1.12)	0.469
Renal disease	1.03 (0.96–1.11)	0.382	–	0.795	**1.30 (1.12–1.51)**	**0.001**	0.98 (0.86–1.13)	0.823
Cardiovascular disease	1.04 (0.97–1.10)	0.283	–	0.263	**1.39 (1.22–1.57)**	**< 0.001**	1.08 (0.97–1.21)	0.175
Asthma or COPD	1.00 (0.95–1.05)	0.985	–	0.220	**1.21 (1.10–1.34)**	**0.001**	1.04 (0.95–1.15)	0.398
Diabetes mellitus	**1.06 (1.02–1.10)**	**0.001**	–	0.643	1.11 (0.99–1.26)	0.077	0.92 (0.82–1.02)	0.117

Bold signals statistically significant p-values (<0.05).

Ref—Reference group, CI—confidence interval, MSM—men who have sex with men, PWID—people who use injection drugs, COPD—chronic obstructive pulmonary disease.

*Model 1 was adjusted for age as of June 1, 2022, sex, race/ethnicity, HIV acquisition risk, highest education level, lowest CD4 count and highest viral load measure over vaccination period, location and substance use.

**Model 2 was adjusted for age as of June 1, 2022, sex, race/ethnicity, HIV acquisition risk, highest education level, lowest CD4 count and highest viral load measure over vaccination period, incarceration history, Body Mass Index and all listed comorbidities including substance use.

^A^Primary series defined as receiving 2 COVID-19 vaccinations, Booster dose defined as receiving 3 or more COVID-19 vaccinations.

^B^Other Ethnicity/Race includes Hispanic, East-Asian, West-Asian, Indo-Asian, or other.

^C^Other HIV risk acquisition group includes blood transfusion, hemophilia or perinatal transmission.

^D^Location defined as most recent place of residence, metropolitan (city of > 1 million people) or non-metropolitan(< 1 million people).

### Characteristics of PWH who had received a COVID-19 booster (3 or more vaccine doses)

**Figure 3:**
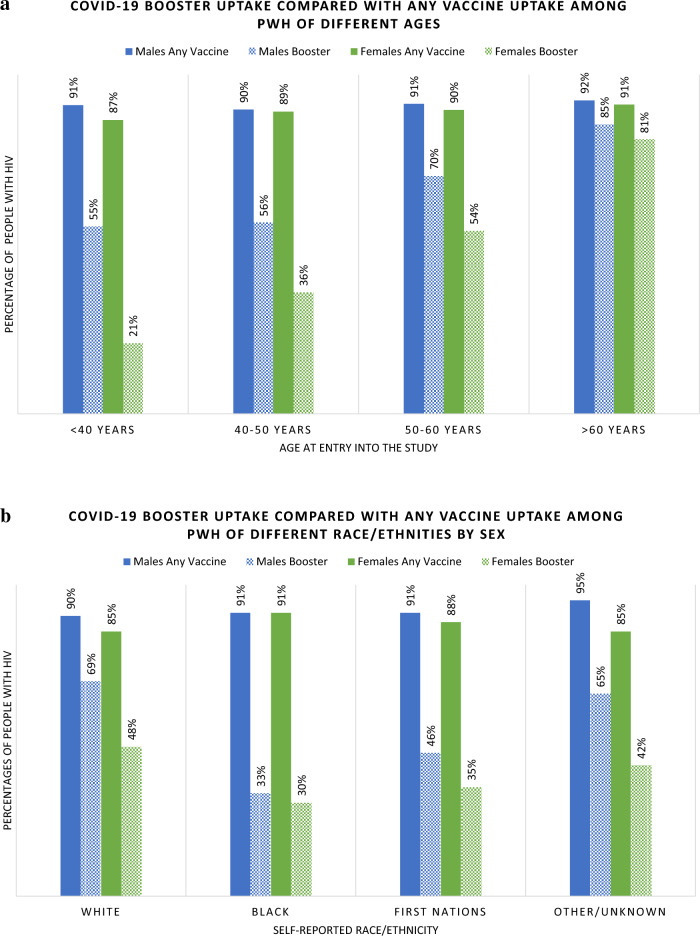
(**a**) COVID-19 booster uptake compared with any vaccine uptake among PWH of different ages. (**b**) COVID-19 booster uptake compared with any vaccine uptake among PWH of different race/ethnicities by sex.

The median age of those who received a booster was 54 years (IQR 44-61) compared to 46 years (IQR 39-53) among PWH who had received only 1–2 doses of vaccine. Among PWH under 50 years, only 38% had received a booster, whereas 62% of those over 50 years received a booster (Table 1). Among males, 60% had received a booster, whereas 36% of females had received a booster. Among all assessed 10-year age groupings, females were less likely to have booster doses, however this difference by sex was greatest among those < 40 years old (Fig. 3a).

Booster uptake was 66% among White participants, 31% among Black participants and 41% among First Nation participants. Among both Black and First Nations PWH, fewer than half who had received at least one dose of COVID-19 vaccine went on to receive a booster dose (Fig. 3b). The majority (73%) of gbMSM PWH received a booster, as compared to 40% of PWH reporting heterosexual HIV acquisition risk and 47% of PWID.

### Factors associated with COVID-19 vaccine booster uptake (vs. 2 dose primary series)

Older age was most strongly associated with booster uptake. Compared to participants < 40 years of age, those aged 40–50 years, 50–60 and > 60 years were 16%, 39% and 61% more likely to have received a COVID-19 booster, respectively (Table 2). The age differential was even more pronounced among female PWH, who demonstrated a 36% (95% CI 1.23–1.50) increased likelihood of booster uptake for each 10-year increase in age, compared to a 12% (95% CI 1.08–1.17) increase with each 10-year increase in age among males.

In unadjusted analyses, females were 38% less likely than males to have received a booster but this association attenuated in adjusted models. Black and First Nations participants were less likely to have received a booster in unadjusted analyses. However, following adjustment this association only remained significant among Black (vs. White) PWH (aPR 0.63 [95% CI 0.54–0.73]). PWH with heterosexual risk activity and PWID were less likely to have received a booster compared with gbMSM in unadjusted analysis, however following adjustment only those with heterosexual risks remained at a 19% lower likelihood of booster uptake (aPR 0.81 [95% CI 0.72–0.91]) when compared with gbMSM PWH.

Housing status, a history of incarceration and location of residency (metropolitan) was not associated with booster uptake. Education level was predictive of booster uptake, with PWH with 12 years of education having a 16% increase in uptake and those with > 12 years having a 20% increase in booster uptake compared to those with < 12 years of education. Substance use was associated with a reduction in booster uptake (aPR 0.90 [95% CI 0.81–0.99]). PWH with CD4 counts of < 200 cells/mm^3^ were 21% (aPR 0.79 [95% CI 0.65–0.96]) less likely to receive a booster compared to PWH with CD4 counts of > 500 cells/mm^3^. PWH with a VL of > 200 copies/mL were 31% less likely to have received a booster (aPR 0.69 [95% CI 0.53–0.91]) compared with PWH with VL $\le$ 200 copies/mL. There was no association between BMI and booster uptake. In bivariate analysis all comorbidities other than diabetes mellitus were associated with a significant increase in booster uptake, however these associations attenuated in multivariable models.

## Discussion

It is important to delineate predictors of COVID-19 vaccine uptake among PWH as prior studies have identified PWH are at greater risk of breakthrough infection^33^ and breakthrough incidence is decreased by receipt of ≥ 3 doses of a SARS-CoV-2 vaccine^34^. In keeping with the “3C” model of vaccine uptake (Complacency, Confidence, Convenience)^11^, we identified correlates of both any vaccine uptake and booster uptake among PWH. While multiple studies, primarily in the early phase of the COVID-19 pandemic, have investigated rates and predictors of VH in PWH^9,35–37^, relatively few have investigated more recent rates of actual vaccine and boosting uptake^8,22^. Our study provides more contemporary evaluation on vaccine uptake among a large geographically defined and heterogeneous cohort of PWH including data from the Omicron era, which began in Canada in November 2021^38^.

Overall, despite the NACI and CDC recommendations, only 1,009 (54%) of the study population had received the recommended third vaccine dose^4,5^. However, during the study period, even fewer (39%) of the Alberta population had received ≥ 3 doses^39^. Similarly, while 10% of our population had received no vaccinations, at the time of the study, approximately 20% of the general Alberta population had not been vaccinated^39^. This increased uptake of vaccine among PWH may in part be related to regular contact with the health care system and care providers, possibly by decreasing complacency (while COVID-19 vaccines were not provided at the clinic, SAC providers regularly encouraged vaccine uptake). Supporting the hypothesis that regular access to care may increase vaccine uptake, we found that PWH who had not had a recently measured CD4 count or HIV VL over the study period were also less likely to receive any COVID-19 vaccination. This is also in keeping with Canada-wide data which demonstrates that those without a regular healthcare provider had lower vaccine uptake^40^.

A prior study among vaccinated PWH found that those with moderate/severe immunodeficiency (CD4 < 350 cells/mm^3^) had a 59% increased risk of severe breakthrough COVID-19 illness (requiring hospitalization) compared with people without HIV^34^. We found that PWH with CD4 counts of < 200 cells/mm^3^ were less likely to receive a COVID-19 booster compared with PWH with CD4 counts > 500 cells/mm^3^. PWH with unsuppressed viral loads (> 200 copies/mL) were also less likely to have received any COVID-19 vaccine or among those who had received a primary series, less likely to have received a booster. This may represent a higher proportion of PWH who are not engaged into HIV care or are socially marginalized, as marginalization has been associated with both lower rates of viral suppression^41^ and COVID-19 vaccination^22^. This highlights the importance of targeted messaging to promote booster uptake.

Supporting the two consistent themes in the VH literature, our analysis found that booster uptake increased with increasing age and decreased with non-White race (specifically among Black PWH). The positive association between age and vaccine uptake aligns with a finding seen across the PWH^9,12,17^ and non-PWH populations^14,23,24^, as well as in studies examining uptake of vaccinations against SARS-CoV-2^8,9,22^ and other vaccine preventable diseases (VPD)^17,21,29^. One study found that the odds of vaccine uptake against SARS-CoV-2 in PWH increased 2.4-fold for each 10-year increase in age^9^, while another demonstrated higher levels of COVID-19 VH in younger age groups^42^. Our analysis found a 22% increase in booster uptake for each 10-year increase in age. The uptake of primary vaccine series, however did not differ by age. This age-dependent difference in booster vs primary series uptake is consistent with broader Alberta-wide data, which as of June 1, 2022 showed only a 4% difference in primary series uptake between 40–59 and $\ge$ 75 years old, but a 34% increase in booster uptake among $\ge$ 75 years old^39^. In Alberta vaccine mandates were introduced between September 2021 and February 2022, mandates only required individuals to receive a primary vaccine series and did not include booster doses. Therefore, it is possible that to maintain work and social activities, younger individuals received a primary series, however, subsequently did not get boosted. Despite not being required by mandates, older individuals were more likely to receive boosting doses potentially for health preservation.

We also found that the age-dependent increase in uptake of COVID-19 booster was differentiated by sex. Younger females were less likely to accept COVID-19 booster compared to males of the same age categories. Prior literature has reported reduced COVID-19 vaccination among young females due to reported concerns around pregnancy, breastfeeding, and fertility^43^. Our study therefore reinforces the need for ongoing efforts addressing vaccine hesitancy targeted to concerns that may differ by both age and sex.

Our observation that vaccine booster uptake was lower in PWH of Black race is similar to that reported in the general VH literature^22,44^. These associations (of age and race) with VH are potentially partly driven by differing levels of confidence (i.e., trust in the medical establishment) and complacency (i.e., perceived vulnerability to VPDs). Some studies examining levels of vaccine confidence have reported higher levels of COVID-19 vaccine-related mistrust among the non-White population^35,45,46^, while older age has been found to be correlated with more perceived vulnerability to VPDs (including SARS-CoV-2) and higher levels of vaccine confidence^14,23,24,47,48^. In contrast to the previous literature, our study found that while Black PWH were less likely to be boosted, they were more likely to have received at least one vaccine compared with White PWH. The reasons for this are unclear, but possibly relate to vaccine mandates, focused messaging campaigns and mobile clinics early in the pandemic targeting communities in Alberta known to have higher proportions of immigrant populations^49,50^, or potentially the intersection between ethnicity and rurality. While recognizing non-White Albertans may be underrepresented in rural areas, we also found that residing in a non-metropolitan area was associated with an increase in the likelihood of receiving no vaccines against COVID-19, although this association attenuated with adjustment.

Relative to gbMSM, heterosexual risk activity and PWID were associated with lower vaccine uptake overall, as well as booster uptake. This replicates previous findings demonstrating that among PWH, MSM had higher vaccine uptake than PWID, both against SARS-CoV-2^22^ and influenza^17,31^. The reasons for this association are not clear, but it may reflect a greater engagement into HIV care among gbMSM PWH. A prior survey of gbMSM demonstrated higher levels of pandemic optimism and therefore greater willingness to accept a COVID-19 vaccine^37^.

Previous studies examining the effect of educational attainment on COVID-19 vaccine uptake have demonstrated a decrease in COVID-19 vaccine uptake among participants who had received less formal education^8,51,52^. Educational attainment has also been found to attenuate racial disparities in COVID-19 vaccine uptake^51,52^. This is likely mediated by differing vaccine confidence, as COVID-19 mistrust beliefs are highest in groups with lower educational attainment^35^. Our findings reinforced this pattern, with increased educational attainment being associated with increased booster uptake. Relatedly, people with low SES and substance use has also been associated with VH, which may be mediated via convenience, as these individuals frequently experience reduced accessibility, limited transportation or other barriers to uptake^53,54^. We also identified a history of substance use was associated with a decrease in booster uptake.

All assessed comorbidities except diabetes were associated with a higher proportion of booster uptake, however in adjusted models these associations attenuated. One possible explanation for this is that age was the driving factor for increased booster uptake and since comorbidities are more common with aging, when adjusted for age, the comorbidity-specific associations were no longer present. As seen in our study, prior studies have documented a reduced uptake of public health preventative measures among people who use substances, including regular health screening, influenza vaccination and COVID-19 vaccination^55–57^. Further efforts are needed to identify barriers to vaccination among people who use substances to reduce this identified gap in uptake.

Strengths of this study include its inclusion of all PWH within a geographically defined area, supported by the centralized HIV care system. However, this study also has limitations. Only data from PWH linked into HIV care in Southern Alberta is included, therefore the conclusions may not be generalizable to other populations. Also, we did not assess for beliefs about vaccination or feelings of vaccine hesitancy, factors which would fall under “Confidence” in the 3C model. As this is an observational and retrospective study, there is likely to be residual confounding. Due to COVID-19, many visits were conducted virtually during the study period leading to unmeasured weights and resulting in 16% missing data for BMI. Factors impacting vaccine uptake but not measured in this study may include political or religious beliefs, healthcare trust, and social media usage. Some of our analyses were limited by small numbers, particularly as we had relatively few participants who had not received any vaccination.

## Conclusion

In Southern Alberta, while PWH have a higher rate of booster uptake against SARS-CoV-2 than the general population, coverage remains suboptimal. The highest risk of not receiving a booster was identified among young and female PWH, as well as those who identified as Black, those who use substances, those with lower educational attainment, lower CD4 counts and unsuppressed HIV viral replication. These findings should prompt more targeted outreach to better understand the unique concerns and to improve vaccine coverage in these high-risk populations. Further research is also needed to investigate the degree to which vaccine hesitancy beliefs play a role in poor booster uptake in these populations, compared to more systemic and geographical factors.

## Data Availability

The datasets generated during and/or analyzed during the current study are not available due to the ethical agreements. Please contact R. Lang (raynell.lang@ahs.ca) regarding access to the deidentified and aggregated datasets generated and/or analysed for this study.
